# The Cardioprotective Potential of Marine Venom and Toxins

**DOI:** 10.3390/toxins18020063

**Published:** 2026-01-26

**Authors:** Virginia Heaven Mariboto Siagian, Rina Fajri Nuwarda

**Affiliations:** 1 Faculty of Pharmacy, Padjadjaran University, Sumedang 45363, Indonesia; virginia22001@mail.unpad.ac.id; 2 Department of Pharmaceutical Analysis and Medicinal Chemistry, Faculty of Pharmacy, Padjadjaran University, Sumedang 45363, Indonesia

**Keywords:** cardiovascular disease, cardiac therapeutics, marine pharmacology, toxin- and venom-derived medicine, cardioprotective agent

## Abstract

Cardiovascular disease (CVD) continues to be the primary cause of morbidity and mortality worldwide, underscoring the urgent need for novel therapeutic alternatives. In recent years, marine ecosystems have garnered increasing attention as a promising source of bioactive compounds with unique structural and pharmacological properties. Marine-derived toxins and venoms, including tetrodotoxin, ω-conotoxins, anthopleurins, palytoxin, brevetoxin, aplysiatoxin, and asterosaponins, exert cardioprotective effects through diverse mechanisms such as modulation of ion channels, inhibition of sympathetic overactivity, antioxidative actions, and enhancement of myocardial contractility. These properties make them potential candidates for addressing various CVD manifestations, including arrhythmia, hypertension, ischemia–reperfusion injury, and heart failure. However, despite their therapeutic promise, the clinical application of these marine compounds remains limited due to poor tissue selectivity, narrow therapeutic indices, proinflammatory activity, and limited metabolic stability. Structural modifications, advanced drug delivery platforms, and in vivo validation studies are crucial for overcoming these challenges. This review highlights the pharmacological actions, molecular targets, and cardiovascular relevance of selected marine toxins and venoms while also addressing key translational barriers. Advances in biotechnology and peptide engineering are enabling the safer and more targeted use of these compounds. Collectively, marine-derived toxins and venoms represent a largely untapped but highly promising frontier in cardiovascular drug discovery. Strategic research focused on elucidating mechanisms, optimizing delivery, and translating clinical applications will be critical to unlocking their full therapeutic potential.

## 1. Introduction

Cardiovascular disease (CVD) is the leading cause of morbidity and mortality globally. According to the World Health Organization (WHO), in 2019, it accounted for 17.9 million deaths annually, representing 32% of all deaths worldwide. CVD encompasses a wide range of heart and blood vessel problems, such as coronary heart disease, heart attack, arrhythmia, heart failure, and several other conditions [1]. Four out of five deaths caused by CVD are related to heart attack and stroke, and one third of these deaths occur early in individuals under the age of 70 [2]. Global analyses likewise show a rising toll of CVD in younger adults between 1990 and 2019. The annual number of CVD deaths among people aged 15–49 increased from ~0.99 to 1.24 million, even as age-specific death rates declined [3]. In other words, heart attacks and strokes increasingly affect young and middle-aged people. This trend implies a significant loss of productive life-years and economic impact, underscoring the need for early interventions targeting risk factors and targeted prevention in younger populations.

Current CVD management relies on conventional therapies, including beta-blockers, angiotensin-converting enzyme inhibitors, antiplatelet agents, and reperfusion interventions. Although these approaches have significantly reduced mortality, their effectiveness is often limited by issues such as drug resistance, adverse effects, and incomplete cardioprotection, particularly in cases of myocardial infarction or heart failure [4,5]. Recognizing these limitations, researchers have turned to alternative sources for new therapeutic agents. The marine environment, which covers more than 70% of the Earth’s surface, harbors extraordinary biodiversity and is increasingly recognized as a promising source of bioactive compounds with diverse structures. Marine organisms from high-biodiversity regions are well known to yield novel bioactive compounds. Besides the Coral Triangle (Indonesia, Philippines, PNG), other marine-rich areas contribute significantly [6]. For instance, extensive exploration of the South China Sea (China) has produced hundreds of new marine metabolites with pharmacological activity. Furthermore, Japanese and Korean coastal waters harbor unique marine species; Japan alone has yielded many extraordinary natural products (e.g., palytoxin, halichondrin) with potent bioactivities. Therefore, countries such as China, Japan, and South Korea with extensive coastlines and diverse marine ecosystems are also important hotspots for marine drug discovery [7].

**Figure 1:**
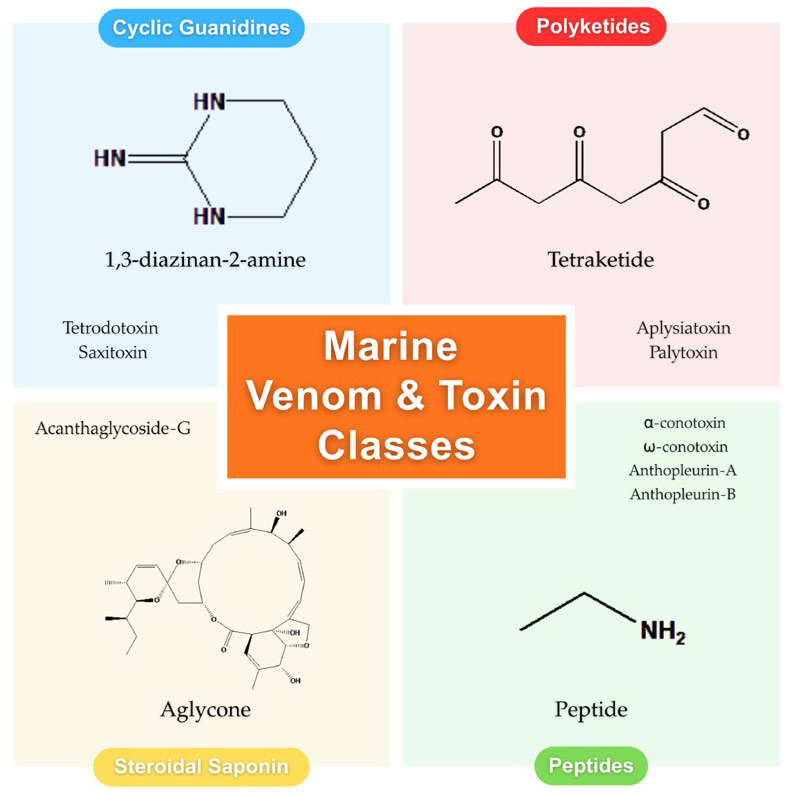
Main structural classes of marine venom and toxin.

Marine-derived compounds often exhibit complex and unique molecular structures, characterized by high stereochemical complexity and the presence of rare functional groups such as halogenated indoles, guanidinium groups, and cyclic imines. These features enable selective and high-affinity interactions with molecular targets, such as ion channels, receptors, and enzymes [8]. Based on their chemical scaffolds, the marine cardiotoxins discussed in this review can be categorized into four main structural classes: cyclic guanidines, polyketides, peptides and saponins, as illustrated in Figure 1.

For instance, ω-conotoxin, a peptide from *Conus magus*, specifically blocks N-type calcium channels and has been approved as a drug. This compound has even been commercialized as the ziconotide drug, which is 100–1000 times more potent than morphine as an analgesic [9]. Similarly, trabectedin, derived from the tunicate *Ecteinascidia turbinata*, targets DNA minor grooves and demonstrates antitumor activity [10]. Marine peptides, such as those isolated from *Holothuroidea* (sea cucumbers), have demonstrated angiotensin-converting enzyme (ACE) inhibition activity, which may contribute to their antihypertensive effects. These discoveries underscore the therapeutic potential of marine toxins and venoms, not only in oncology and neurology, but also in cardiovascular medicine [11].

Although toxins and venoms from marine organisms are frequently associated with adverse effects, such as neurotoxicity, cardiotoxicity, or cytotoxicity, these properties often arise from their high specificity and potency in targeting critical physiological systems, including ion channels, enzymes, and receptors [12]. However, recent research has shifted the perspective on these bioactive molecules, recognizing their therapeutic potential in the development of new drugs. Several marine-derived toxins have shown promise in preclinical and clinical studies for diverse medical applications. Conantokin-G has demonstrated anticonvulsant effects by acting as an NMDA receptor antagonist, and micropeptins from cyanobacteria have shown DPP-IV inhibition relevant to diabetes management [13,14].

In contrast to previous reviews that broadly categorize marine natural products, this work specifically provides a critical analysis of the molecular mechanisms, particularly ion channel modulation, underlying the cardioprotective effects of marine toxins. Furthermore, we integrate recent data connecting their mechanisms of activity with their structural chemistry and highlight the translational gap between preclinical success and clinical application, offering a novel perspective on overcoming stability and toxicity issues.

## 2. Methods

A literature search was conducted using PubMed databases to identify studies published between 2015 and 2025. The search employed keywords such as “marine toxins,” “venoms,” “cardioprotective,” and “marine pharmacology.” Inclusion criteria were limited to peer-reviewed articles published in English that reported original data on the cardiovascular effects of marine-derived compounds. Studies lacking full-text availability or those focusing solely on non-cardiovascular bioactivities were excluded. This review prioritizes studies that provide mechanistic insights into the cardioprotective potential of these compounds.

## 3. Marine Toxins and Venoms

Biochemically, marine toxins and venoms are distinct entities. Toxins are typically accumulated metabolites (often dietary) that enter the human body through the consumption of contaminated seafood or environmental exposure. In contrast, venoms are complex mixtures of bioactive compounds that are actively delivered into prey or predators via specialized apparatuses such as stingers, fangs, or spines. Despite these delivery differences, both groups possess potent biological activities targeting the nervous and cardiovascular systems. Based on their physicochemical properties, these compounds are broadly categorized into hydrophilic (water-soluble), lipophilic (fat-soluble), and amphipathic classes. This polarity dictates their bioavailability, distribution, and mechanism of action within the host. Table 1 summarizes the biochemical classification of these major marine compounds alongside their lethal toxicity profiles (LD_50_).

An analysis of the toxicological data presented in Table 1 reveals a significant disparity in potency across different biochemical classes. Compounds targeting voltage-gated ion channels, such as CTX and TTX, exhibit extreme lethality with LD_50_ values in the microgram range (<1 µg/kg). This high potency is attributed to their high-affinity binding to critical ion channels (e.g., NaV1.5) where even picomolar concentrations can disrupt electrochemical gradients essential for cardiac and neuronal function. In comparison, amino acid derivatives like DA demonstrate relatively lower acute toxicity (LD_50_ in the milligram range), suggesting a different threshold for lethality. Furthermore, the presence of amphipathic compounds such as PLTX, which possesses dual solubility properties, allows for unique interactions with cell membranes, resulting in catastrophic disruption of ion homeostasis. This toxicological profile highlights a critical challenge in drug discovery: while high-potency toxins offer precise molecular targeting for cardiac conditions, their narrow therapeutic index requires rigorous optimization to balance efficacy against the risk of lethal toxicity.

## 4. Cardioprotective Mechanisms of Marine Toxin Compounds

Toxic compounds produced by marine organisms have therapeutic potential in various cardiovascular diseases. These compounds work through various mechanisms, including ion channel modulation, inhibition of calcium channels, inotropic support, reperfusion therapy, and anti-inflammatory and antioxidant properties. The toxic compounds produced by these marine organisms show various similarities and differences in chemical structure, mechanism of action, and potential cardioprotective effects. One of the main similarities is their interaction with ion channels, which play a crucial role in the electrophysiological function of the heart [43,44,45].

Additionally, these toxic compounds exhibit significant differences in type and chemical structure. Conotoxins and TTX are peptides and small alkaloids, respectively, while PLTXs from *Palythoa* spp. and asterosaponins from *Acanthaster planci* are large molecules with complex chemical structures. Palytoxin is one of the world’s most toxic non-protein compounds, acting on sodium/potassium ion pumps to cause extreme cell depolarization. Meanwhile, asterosaponins, which belong to the triterpenoid glycoside family, have a distinct mechanism with anti-inflammatory and lipid-regulating potential that may aid in the prevention of cardiovascular diseases more broadly [46]. Another difference lies in the distribution of these compounds in nature. Some species, such as *Conus magus* and *Anthopleura* spp., produce toxins that are injected into their prey through specialized structures, including proboscises and nematocysts [47]. In contrast, some other species, such as *Takifugu rubripes* and *Astropecten latespinosus,* contain toxins that accumulate in body tissues, including the liver and skin, which are harmful when consumed [48]. The links between marine toxic compounds and various heart diseases are discussed below.

### 4.1. Ion Channel Modulation

Ion channel modulation represents an effective therapeutic strategy in cardiovascular pharmacology as ion channels are essential in generating and regulating action potentials in cardiac myocytes. By influencing the flow of ions such as sodium, potassium, and calcium across the cardiac cell membrane, these channels directly control heart excitability and rhythm. Disruption to ion channel function can lead to electrophysiological abnormalities, manifesting as arrhythmia, a condition characterized by irregular heart rhythms due to impaired impulse formation or conduction in the myocardium. Common causes of arrhythmia include ion imbalances, structural cardiac changes, and adverse drug reactions. Several toxic compounds derived from marine organisms have demonstrated the ability to modulate ion channel activity by selectively inhibiting specific ion pathways, offering potential as novel antiarrhythmic agents [49]. For instance, TTX binds to voltage-gated sodium channels (NaV) and blocks the fast inward sodium current (*I*_Na_) that underlies rapid depolarization (phase 0) of the cardiac action potential. This inhibition prevents abnormal excitability and stabilizes electrical conduction, making TTX a potential candidate for managing certain types of ventricular arrhythmias [50]. However, the modulation of sodium channels is not limited to pore blockade; distinct mechanisms that alter channel gating kinetics can also yield significant cardioprotective effects.

In this context, sea anemones (*Cnidaria*) produce a rich array of polypeptide toxins known as anemonotoxins, which are potent modulators of NaV. Among these, ATX II, isolated from the snakelocks anemone (*Anemonia sulcata*), is of relevance to cardiovascular physiology. Unlike TTX, which occludes the channel pore, ATX II acts by delaying the inactivation of cardiac sodium channels (NaV_1.5_). Mechanistically, ATX II binds to the channel’s extracellular loop, preventing the conformational change required for fast inactivation. This results in a persistent sodium current (*I*_NaL_), leading to a prolonged action potential duration (APD) and an increase in intracellular calcium via the Na^+^/Ca^2+^ exchanger. While this mechanism is often used to model Long QT Syndrome type 3 (LQT3) and arrhythmias in research, it also highlights a potential therapeutic pathway: controlled enhancement of cytosolic calcium can exert a positive inotropic effect [51]. Thus, modified derivatives of anemonotoxins are being investigated as potential leads for treating heart failure, provided that their pro-arrhythmic risks can be minimized.

These bioactive compounds have attracted interest in their potential use as cardioprotective agents due to their ability to modulate specific molecular targets. Various marine toxins and venoms with cardioprotective potential, their source species, toxic compounds, and classification as either toxins or venoms in the cardiovascular system are listed in Table 2.

**Figure 2:**
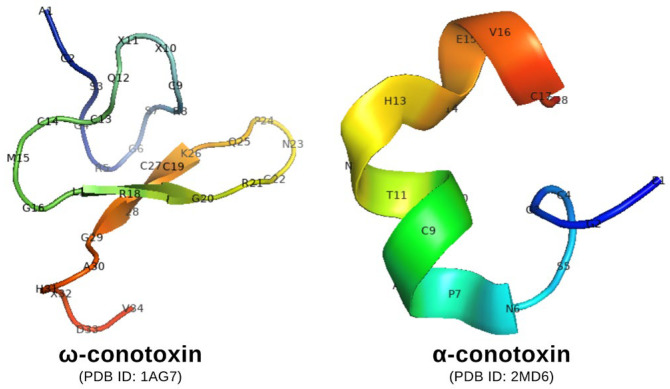
Chemical structure of ω- and α-conotoxin.

In parallel, ω-conotoxin MVIIA, a peptide derived from *Conus magus*, selectively targets N-type calcium channels (CaV2.2), which are predominantly located on sympathetic nerve terminals. Inhibition of these channels reduces calcium influx, thereby suppressing the release of norepinephrine, a key mediator of sympathetic overactivity. Through this mechanism, ω-conotoxin may attenuate excessive autonomic stimulation, thereby contributing to the control of blood pressure and rhythm disturbances [70,71]. Complementing this sympathetic inhibition, α-conotoxins target the opposing parasympathetic pathway by antagonizing nicotinic acetylcholine receptors (nAChRs). This distinct mechanism modulates vagal tone, providing a comprehensive toolkit for restoring cardiac autonomic balance [72]. The structural features of representative α- and ω-conotoxins that underlie these functional differences are illustrated in Figure 2.

**Figure 3:**
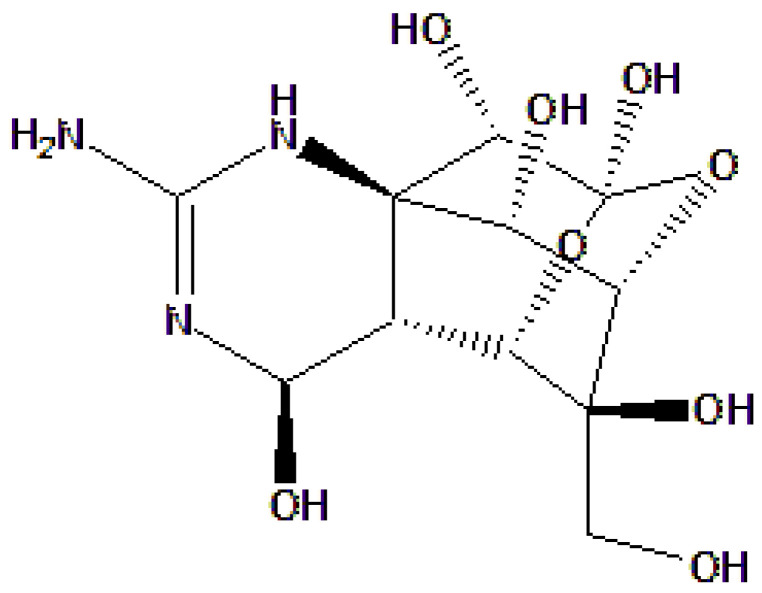
Chemical structure of tetrodotoxin (TTX).

The mentioned instances highlight the mechanism of targeting specific ion channels, sodium channels in cardiac muscle cells, and calcium channels in autonomic neurons that enable precise modulation of cardiac excitability and neurohormonal signaling. These mechanisms offer promising alternatives for the development of antiarrhythmic agents derived from marine toxins. Figure 3 shows the chemical structure of TTX, highlighting its highly oxygenated, guanidinium-containing scaffold.

TTX is a potent marine neurotoxin characterized by a highly oxygenated tricyclic scaffold bearing a guanidinium moiety, which mimics hydrated Na^+^ and mediates high-affinity binding to NaV channels. These channels are essential membrane proteins that open in response to depolarization, allowing sodium ions to enter cells that are excitable, such as neurons and cardiomyocytes. TTX can inhibit the NaV1.4, NaV1.6, and NaV1.7 subtypes with remarkable potency and selectivity, thereby preventing the *I*_Na_ responsible for rapid depolarization. Through blocking Na^+^ influx into cardiomyocytes, TTX suppresses overexcitation and lowers the risk of ventricular tachycardia and fibrillation, thereby stabilizing the cardiac rhythm, especially in patients with long QT syndrome or sympathetic hyperactivity-induced arrhythmias [73]. Moreover, structure–activity relationship (SAR) studies confirmed that alterations to TTX’s guanidinium or hydroxyl groups markedly reduce its channel affinity, underscoring the critical role of its precise chemical structure [50,74].

### 4.2. Inhibition of Calcium Channel Blockers (CCBs)

Inhibition of calcium channels plays a crucial role in cardiovascular therapy. While L-type blockade is commonly used for vasodilation and blood pressure management, targeting N-type calcium channels offers a different cardioprotective pathway. These channels are targeted by ω-conotoxins, neurotoxic peptides derived from the venom of *Conus* spp., such as *Conus magus* and *Conus striatus*. Among marine peptides, ω-conotoxins isolated from *Conus magus* are distinct for their highly specific action on voltage-gated calcium channels. Structurally, these are 25–27 amino acid peptides stabilized by three disulfide bridges, forming a rigid “inhibitor cystine knot” motif that confers high stability and selectivity. These channels open in response to membrane depolarization (threshold ≈ −40 mV) and permit Ca^2+^ influx (*I*_Ca_) into presynaptic sympathetic nerve terminals. Inhibiting these channels diminishes calcium-triggered exocytosis of norepinephrine and acetylcholine, thereby attenuating sympathetic overstimulation, which can underlie atrial fibrillation and stress-related tachyarrhythmias [75]. Conversely, brevetoxin modulates NaV channels by prolonging their open state, thereby increasing initial excitation while preventing excessive repolarization that leads to arrhythmias. Such a dual effect may stabilize myocardial excitability under hypoxic or ischemic conditions [76].

**Figure 4:**
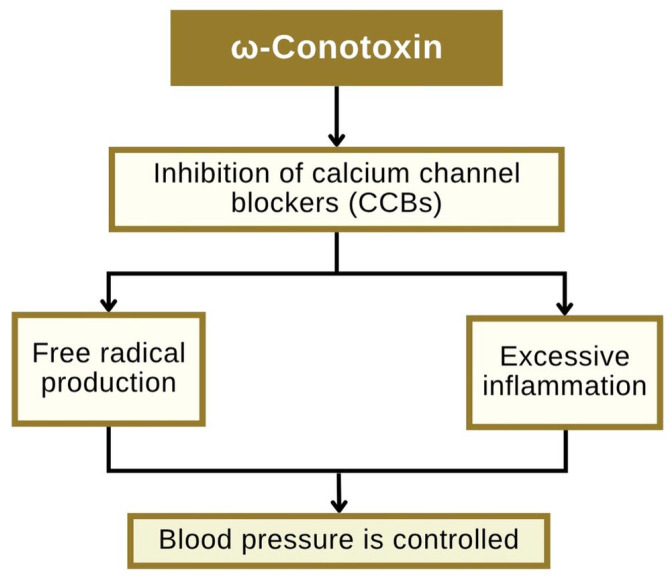
Diagram of the mechanism of ω-conotoxin’s inhibition of hypertension.

Hypertension, or elevated blood pressure, is a major risk factor for coronary artery disease and stroke. Prolonged hypertension contributes to vascular endothelial damage, dysregulation of blood pressure control systems, and chronic inflammation, processes that can lead to atherosclerosis and reduced myocardial perfusion [77]. Blood pressure regulation is physiologically governed by vascular tone, blood volume, and neurohormonal control. Endothelial dysfunction, often present in individuals with hypertension, results in an imbalance between vasodilators, such as nitric oxide (NO), and vasoconstrictors, including angiotensin II [78]. Therefore, targeting calcium channels and restoring endothelial function represent key therapeutic approaches for the treatment of hypertension and coronary artery disease. Figure 4 illustrates the mechanism through which ω-conotoxin exerts its antihypertensive effect: selective inhibition of CaV2.2, followed by a reduction in calcium entry and neurotransmitter release.

The downstream effects include decreased stimulation of NADPH oxidase, resulting in reduced production of reactive oxygen species (ROS) and diminished vascular inflammation [79]. The mechanism differs from that of conventional calcium channel blockers, such as amlodipine, which directly inhibit L-type channels in vascular smooth muscle. In addition, the more specific therapeutic effect of ω-conotoxins lies in their selective inhibition of CaV2.2 channels within the autonomic nervous system, allowing for targeted suppression of excessive sympathetic stimulation that often occurs in hypertensive patients without directly altering vascular smooth muscle tone [80]. This mechanism holds promise in individuals whose hypertension is primarily driven by autonomic overactivity.

Research on *Acanthaster planci*, a crown-of-thorns starfish found in Vietnamese waters, led to the isolation of a novel asterosaponin called acanthaglycoside G, along with three previously known steroidal pentaglycosides. Acanthaglycoside G is classified as a rare asterosaponin due to its carbohydrate composition, which includes only two specific 6-deoxymonosaccharide units: β-D-fucopyranosyl and β-D-quinovopyranosyl. These sugar residues are structurally similar to those found in plant-derived cardiac glycosides such as digoxin and digitoxin, which interact with Na^+^/K^+^-ATPase and exert positive inotropic effects on the cardiovascular system [81].

**Figure 5:**
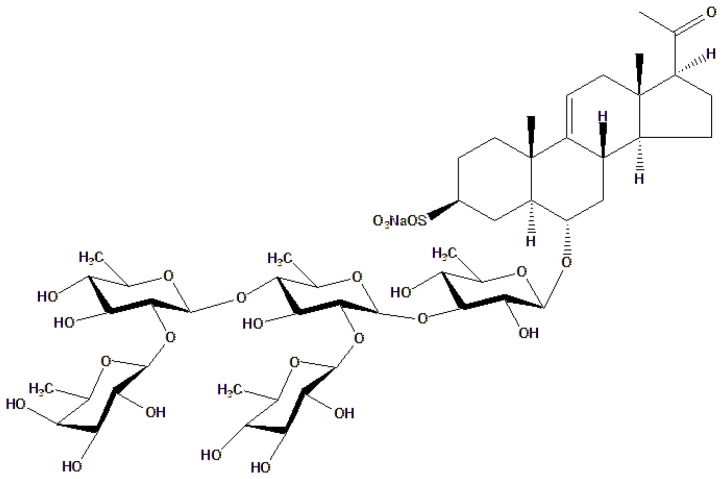
Chemical structure of acanthaglycoside-G.

However, the pharmacological activity of acanthaglycoside G cannot be attributed solely to the sugar content. Rather, its biological potential arises from the synergistic contribution of both the steroidal aglycone and glycosidic moieties. The sugar residues modulate solubility, receptor binding, and pharmacokinetic behavior, while the aglycone provides the core pharmacophore that likely interacts with Na^+^/K^+^-ATPase. SAR studies on similar glycosides have demonstrated that even minor alterations in sugar composition can significantly impact therapeutic efficacy [82]. Figure 5, which presents the chemical structure of acanthaglycoside G, highlights these functionally important glycosidic linkages.

The specific structural configuration of asterosaponins from *Acanthaster planci*, which includes a cardiotonic steroid aglycone core linked to selectively positioned oligosaccharides, supports the hypothesis that these compounds may act through mechanisms similar to those of classical cardiac glycosides. The combination of the steroid nucleus and the unique sugar moieties enables interaction with cardiac ion transporters, such as Na^+^/K^+^-ATPase, and contributes to the modulation of cardiovascular function. In addition, only two other asterosaponins with structurally comparable features have been reported to date: archasteroside B and C from the tropical starfish *Archaster typicus*, as described by Kicha et al. [83]. Further research into the structural diversity and bioactivity of asterosaponins from echinoderms could potentially pave the way for the development of novel cardioprotective agents.

### 4.3. Inotropic Support

Inotropic support plays a crucial role in managing impaired cardiac contractility by enhancing myocardial contraction force, thereby improving cardiac output in conditions where the heart struggles to meet circulatory demands. This mechanism is particularly relevant in heart failure, a condition in which the heart loses its ability to pump sufficient blood to the tissues, resulting in compromised perfusion and metabolic dysfunction. Heart failure typically begins with damage to the myocardium, followed by a decline in cardiac output. When the body’s metabolic demands are not met, the heart activates various compensatory mechanisms, including neurohormonal activation and structural remodeling, to maintain adequate perfusion. Structural remodeling refers to the long-term morphological and functional changes in cardiac tissue, including alterations in chamber size, wall thickness, fibrosis, and myocyte hypertrophy. While initially adaptive, these changes ultimately contribute to the progression of heart failure by reducing cardiac efficiency and increasing oxygen demand [84].

**Figure 6:**
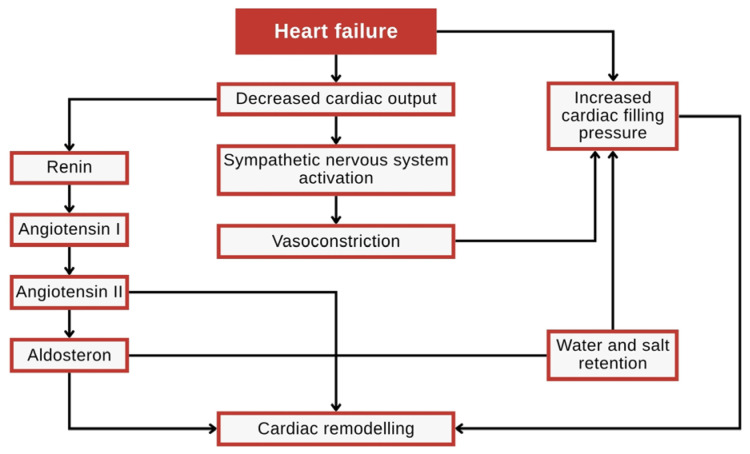
Diagram of heart failure mechanisms.

However, these adaptations, such as increased preload, afterload, and ventricular hypertrophy, can further exacerbate cardiac dysfunction over time. Increased preload, the volume of blood returning to the heart at the end of diastole, and elevated afterload, the resistance the heart must overcome during systolic ejection, are among the hemodynamic stressors that trigger these maladaptive responses. Chronic elevation of preload and afterload leads to ventricular dilation and hypertrophy, which impair contractility and promote further neurohormonal dysregulation [85]. One therapeutic approach for heart failure is to enhance cardiac contractility with positive inotropic agents, which can increase the heart’s pumping force without excessively increasing oxygen consumption. In this regard, several toxic compounds produced by marine organisms have shown potential as effective inotropic agents. Figure 6 illustrates the pathophysiological cycle of heart failure, highlighting how reduced cardiac output activates compensatory mechanisms, including sympathetic stimulation, fluid retention, and myocardial remodeling.

Over time, these compensations become detrimental, leading to worsening cardiac function, congestion, and systemic symptoms. The diagram serves as a visual summary of the interplay between hemodynamic burden, neurohormonal activation, and structural remodeling that underlie the progression of chronic heart failure.

**Figure 7:**
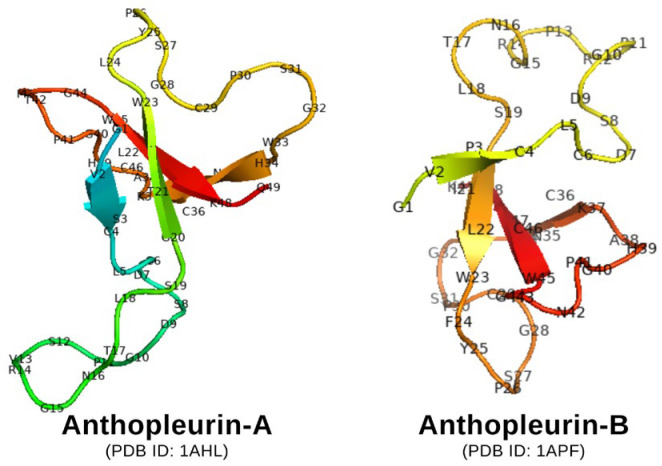
Chemical structure of anthopleurin-A and B.

Anthopleurin-A and anthopleurin-B, two peptide compounds found in *Anthopleura* spp., work by increasing the sensitivity of sodium channels in myocardial cells. This mechanism prolongs the action potential and increases cardiac contractility without increasing the risk of significant arrhythmias, in contrast to digitalis, which can trigger heart rhythm. Figure 7 shows the chemical structure of anthopleurin-A and B.

**Figure 8:**
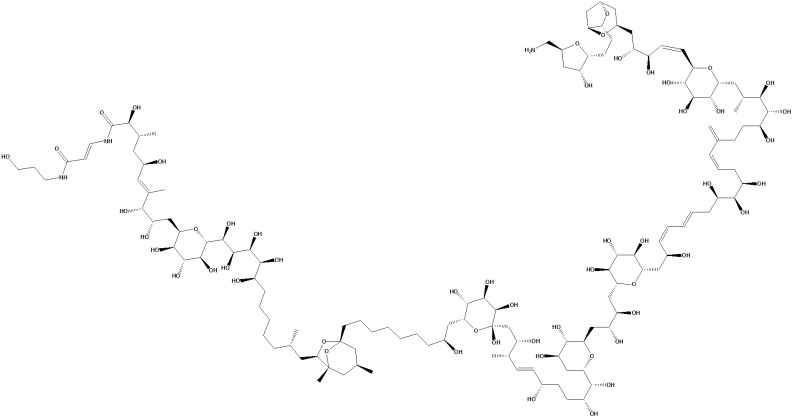
Chemical structure of PLTX.

The positive inotropic effects of these compounds make them potential candidates in heart failure therapy, especially for conditions with systolic dysfunction. In addition, PLTXs from *Palythoa* spp. also exhibit positive inotropic activity through a different mechanism. This compound works by modulating the sodium/calcium ion pump (Na^+^/K^+^ ATPase), which in turn increases the sodium and calcium ion gradients within myocardial cells [86]. However, despite its therapeutic potential, PLTX has high toxicity and a narrow therapeutic range, so further research is needed to adjust the dose and reduce its side effects. Therefore, further research is still needed to explore how these compounds can be modified or used in safer heart failure therapy formulations. Figure 8 shows the chemical structure of PLTX.

### 4.4. Reperfusion Therapy

**Figure 9:**
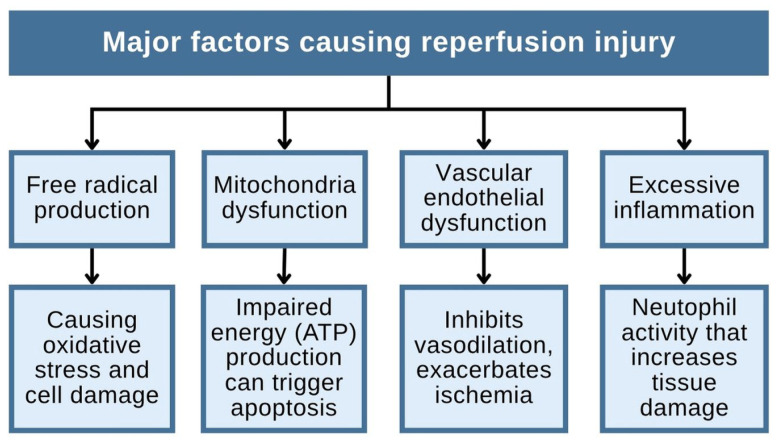
Diagram of the main factors causing reperfusion injury.

Reperfusion therapy, such as coronary angioplasty or thrombolysis, aims to restore blood flow to heart tissue that has been deprived of oxygen due to an obstruction in a coronary artery. While this intervention is essential to limit myocardial damage, it can paradoxically lead to reperfusion injury, a phenomenon characterized by oxidative stress, inflammation, and metabolic disturbances triggered by the sudden reintroduction of oxygen [87]. These pathological processes can further harm the myocardium. Such therapy is particularly relevant in the context of a heart attack or acute myocardial infarction (AMI), which occurs when a coronary blockage causes myocardial ischemia. Without prompt treatment, the oxygen-deprived cardiac tissue progresses to necrosis or irreversible cell death. Reperfusion injury is tissue damage that occurs when blood flow returns to the heart after a period of ischemia. This process is influenced by several major factors, such as free radical production, mitochondrial dysfunction, vascular endothelial dysfunction, and excessive inflammation [88]. Figure 9 illustrates these four interrelated mechanisms: (1) an explosion of ROS that causes oxidative stress and direct cell damage; (2) mitochondrial dysfunction, which disrupts ATP production and triggers apoptosis; (3) vascular endothelial dysfunction, which limits vasodilation and exacerbates ischemia; and (4) excessive inflammation, particularly neutrophil activation, which exacerbates tissue damage through the release of proteases and ROS [89]. Combined, these processes exacerbate myocardial damage even after blood flow has been restored.

**Figure 10:**
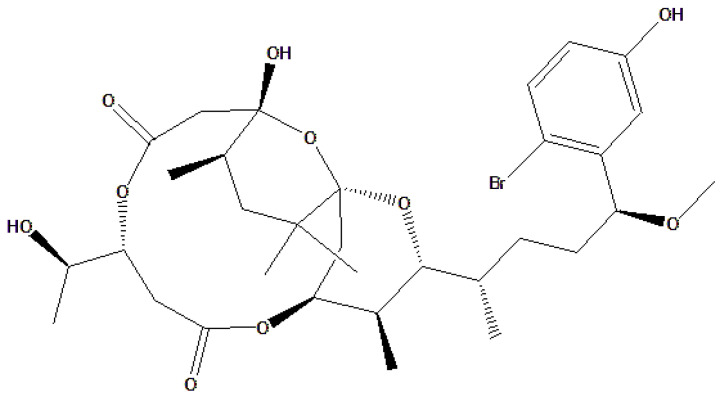
Chemical structure of aplysiatoxin.

In this context, some marine toxic compounds, such as TTX and aplysiatoxin, may have protective potential against reperfusion injury through specific mechanisms related to these factors. During reperfusion, increased calcium ions may stimulate free radical production in mitochondria. By reducing calcium accumulation, TTX may help mitigate oxidative stress and tissue damage caused by ROS [90]. Aplysiatoxin is a toxic compound produced by the cyanobacteria *Lyngbya* spp. that lives in marine ecosystems. Aplysiatoxin is known to have proinflammatory activity and tumor-promoting properties through activation of protein kinase C (PKC). However, studies have developed simple analogs (of aplysiatoxin) that exhibit antiproliferative activity against several cancer cell lines without significant proinflammatory effects. One such analog is 10-methyl-aplog-1. The development of 10-methyl-aplog-1 shows potential in suppressing excessive inflammation. This suggests that modification of the chemical structure of aplysiatoxin may reduce its proinflammatory effects, enhancing its therapeutic activity [91,92]. Figure 10 shows the chemical structure of aplysiatoxin.

## 5. Advances and Obstacles in Cardiovascular Drug Development

### 5.1. Development of Toxic Compounds as Cardiovascular Drugs

Marine toxins and venoms have been recognized for over half a century as a rich source of biologically active compounds with highly specific mechanisms of action. Since the 1960s, compounds such as tetrodotoxin and saxitoxin have been extensively studied for their ability to modulate ion channels. Through cardiovascular research, several peptides and small molecules derived from marine and terrestrial venoms have been shown to exhibit promising cardioprotective properties, including anti-arrhythmic effects, protection against ischemia–reperfusion injury, and blood pressure modulation [93]. These venom-derived compounds often exert their effects by modulating critical cardiac targets, such as ion channels, enzymes, or receptors, in ways that differ from those of conventional drugs. For instance, snake venom natriuretic peptides act via natriuretic peptide receptors to induce vasodilation and cGMP-mediated cardioprotection, while diverse peptide toxins selectively modulate voltage-gated NaV, CaV, and KV channels with greater specificity than traditional small-molecule blockers [94,95]. While some venom-derived agents, such as captopril, originally developed from *Bothrops jararaca* snake venom peptides, have been approved for cardiovascular treatment, only a few marine-based candidates have progressed beyond preclinical evaluation. To date, several marine compounds have demonstrated cardioprotective properties, but most remain in experimental stages, with only limited examples undergoing clinical investigation or being conditionally approved for non-cardiac uses [96,97,98]. Table 3 summarizes the main toxins under investigation for cardioprotection.

#### 5.1.1. ω-Conotoxins

ω-Conotoxins are peptide neurotoxins that selectively inhibit CaV2.2 channels, which are predominantly expressed in neuronal tissues. These channels play a crucial role in regulating the release of neurotransmitters, particularly glutamate, at presynaptic terminals. By blocking CaV2.2 channels, ω-conotoxins significantly reduce calcium influx into neurons, thereby inhibiting the exocytosis of excitatory neurotransmitters and attenuating excitotoxicity, a pathological process implicated in chronic pain and neurodegenerative disorders. For instance, ω-conotoxin Bu8, isolated from *Conus bullatus*, demonstrated potent analgesic activity with fewer motor side effects compared to the clinically used ω-conotoxin MVIIA (ziconotide) [106].

**Figure 11:**
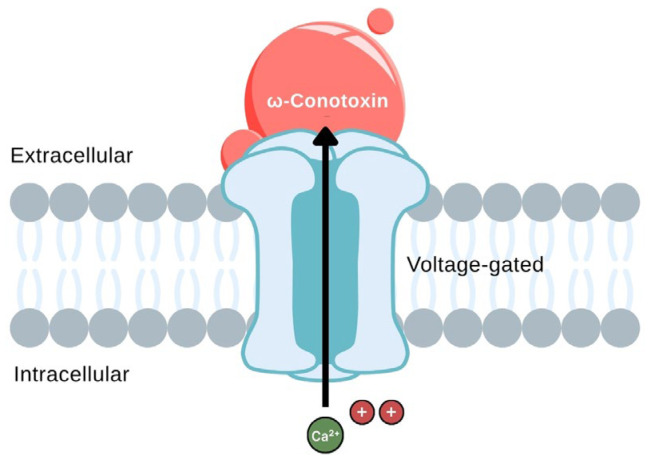
Mechanism of action of ω-conotoxin GVIA on the CaV2.2 channel. The red molecule represents ω-conotoxin blocking the calcium channel pore. The large black arrow pointing upward indicates the inhibition of Ca^2+^ ion influx into the intracellular space. (The figure was adapted from Jobling [108].)

Additionally, α-conotoxin Vc1.1 has been shown to suppress CaV2.2 channel activity indirectly through GABA_B_ receptor activation and downstream Gi/o protein signaling, offering a mechanistically distinct pathway from direct channel blockade. Molecular dynamics simulations have further elucidated how ω-conotoxin GVIA interacts with the CaV2.2 channel, revealing key structural domains involved in its high-affinity binding and selectivity [107]. Figure 11 illustrates the extracellular binding of ω-conotoxin GVIA at the pore entrance of the channel, effectively acting as a physical barrier to calcium ion entry. This spatial blockade prevents depolarization-induced Ca^2+^ influx into sympathetic nerve terminals, thereby reducing neurotransmitter release and dampening sympathetic overactivity. The highly specific interaction depicted in the figure underscores the value of ω-conotoxins as precise modulators of autonomic neurotransmission, with potential cardiovascular applications.

#### 5.1.2. Conantokin-G

**Figure 12:**
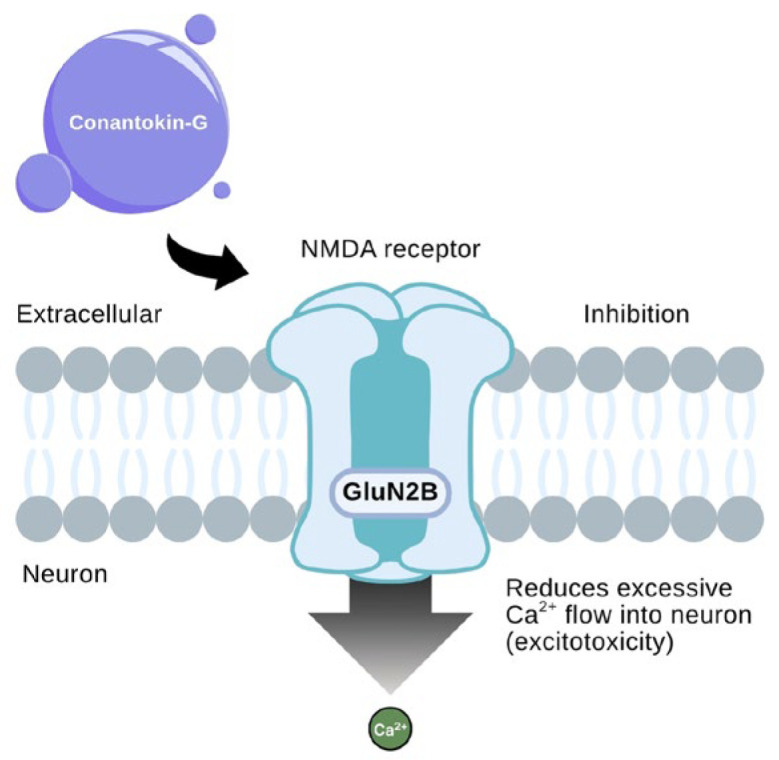
Mechanism and neuroprotective effects of conantokin-G’s action on NMDA receptors. The purple sphere represents conantokin-G acting as an NMDA receptor antagonist. The large downward gradient arrow illustrates the reduction of excessive Ca^2+^ flow into the neuron to prevent excitotoxicity. (The figure was adapted from Mody & McDonald [110].)

Conantokin-G (Con-G), a peptide derived from *Conus geographus* venom, exerts its neuroprotective effects by selectively inhibiting N-methyl-D-aspartate receptors (NMDARs), particularly those containing the GluN2B subunit. As shown in Figure 12, Con-G binds extracellularly to the NMDA receptor complex on neuronal membranes, resulting in the inhibition of receptor activity. This blockade reduces the excessive influx of calcium ions (Ca^2+^) into neurons, a critical step in preventing excitotoxicity, a major contributor to neuronal death during ischemic stroke [109].

By mitigating this pathological calcium overload, Con-G significantly reduces infarct size and promotes neuronal survival. Additionally, the GluN2B-selectivity of Con-G enhances its safety and efficacy profile by limiting the widespread inhibition of NMDA receptors, which is often associated with severe side effects. This mechanism also highlights its potential applicability beyond cerebral ischemia, such as in cardiac ischemic injury, where glutamate-mediated excitotoxic pathways similarly contribute to cell death. Figure 12 illustrates how conantokin-G binds selectively to the GluN2B subunit of the NMDA receptor, located on the extracellular domain of neuronal membranes [111]. This interaction sterically hinders the receptor’s calcium-permeable pore, thereby attenuating the pathological influx of Ca^2+^ ions during excitotoxic events. While NMDA receptors are primarily associated with central nervous system activity, their involvement in cardiac ischemia-related neuronal injury has become increasingly evident. The figure highlights how targeted modulation of GluN2B-containing NMDA receptors may offer a dual neuro- and cardioprotective benefit by reducing calcium overload and subsequent oxidative stress [110].

#### 5.1.3. Tetrodotoxin (TTX)

TTX is a potent neurotoxin known for its high affinity and selectivity in blocking NaV channels, particularly the NaV1.1–NaV1.7 subtypes, by binding to the extracellular pore region of the α-subunit. This binding prevents sodium influx during the depolarization phase of the action potential, thereby inhibiting electrical conduction in excitable tissues such as cardiac myocytes. In the context of ventricular arrhythmias, TTX’s selective inhibition of Na^+^ channels can suppress abnormal automaticity and conduction reentry circuits, both of which are central to the pathophysiology of these arrhythmias [112].

**Figure 13:**
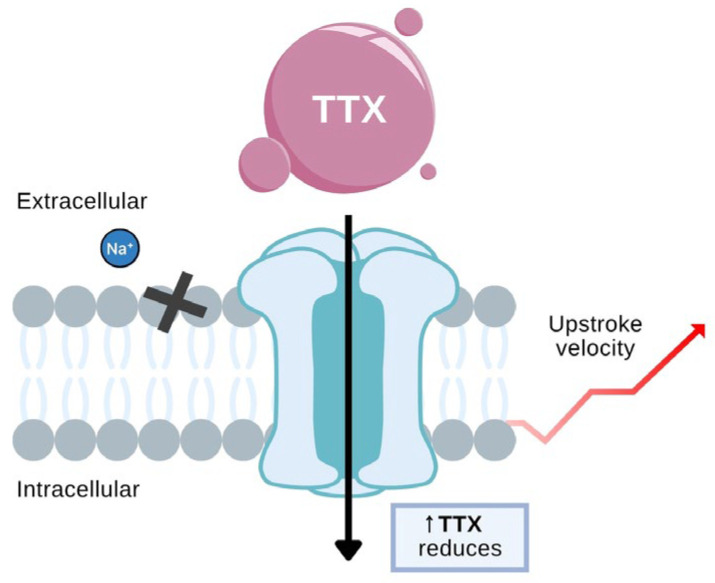
Mechanism of tetrodotoxin (TTX)’s action on NaV channels. The black cross (X) represents the blockade of Na^+^ ion entry by TTX, and the red arrow indicates the upstroke velocity of the action potential. (The figure was adapted from Nieto et al. [115].)

As illustrated in Figure 13, TTX binds to the extracellular vestibule of the NaV channel, forming a physical block that halts Na^+^ entry into the cell. This inhibition prevents rapid depolarization and decreases the upstroke velocity of the cardiac action potential. By stabilizing the channel’s inactive conformation, TTX reduces the likelihood of repetitive or ectopic excitation, key contributors to arrhythmogenesis. The visual emphasizes how reduced sodium influx underlies TTX’s electrophysiological suppression of abnormal cardiac impulses. Recent studies suggest that low doses of TTX can selectively target the late sodium current (*I*_NaL_), which is often upregulated in diseased myocardium, without significantly impairing normal conduction, highlighting its therapeutic potential in drug-resistant ventricular arrhythmias [113,114].

#### 5.1.4. Bradykinin-Potentiating Peptides (BPPs)

Although most clinically approved cardiovascular drugs derived from venomous species, such as captopril, originate from terrestrial sources like snake venom-derived BPPs, marine toxins represent a comparatively underexplored yet highly promising reservoir of bioactive compounds with therapeutic potential. BPPs and their synthetic analog captopril exert their antihypertensive effects predominantly by inhibiting ACE, a key regulator of the renin–angiotensin–aldosterone system (RAAS). ACE facilitates the conversion of angiotensin I to angiotensin II, a potent vasoconstrictor that mediates increased systemic vascular resistance, promotes sodium and water retention, stimulates sympathetic nervous activity, and induces pathological cardiac remodeling. Pharmacological inhibition of ACE leads to decreased angiotensin II bioavailability, thereby mitigating vasoconstriction and aldosterone-driven volume overload, ultimately resulting in blood pressure reduction [116]. In parallel, ACE inhibition enhances the endogenous levels of bradykinin by preventing its degradation. Elevated bradykinin concentrations enhance endothelial nitric oxide and prostacyclin synthesis, thereby contributing to vasodilation and the attenuation of vascular inflammation and fibrosis. This dual mechanism suppression of angiotensin II and potentiation of bradykinin underpins the therapeutic efficacy of ACE inhibitors not only in hypertension management, but also in delaying the progression of cardiac hypertrophy and fibrosis [117].

**Figure 14:**
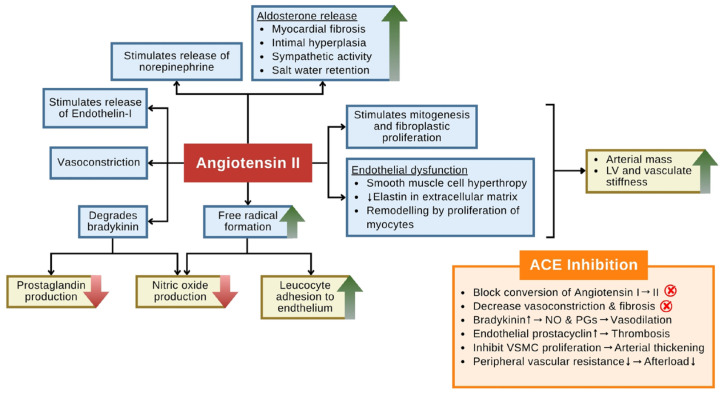
Pathophysiological effects of angiotensin II and the therapeutic role of ACE inhibitors in cardiovascular disease. Green upward arrows indicate an increase in activity or production (e.g., free radical formation and aldosterone release). Red downward arrows represent a decrease in production (e.g., nitric oxide). Red cross signs in circles mark the specific points where ACE inhibition interrupts the pathway. (The figure was adapted from Borghi et al. [119].)

Figure 14 illustrates the multifactorial pathophysiological roles of angiotensin II in promoting cardiovascular dysfunction and highlights the counter-regulatory effects of ACE inhibition. Angiotensin II, a central effector peptide of the renin–angiotensin–aldosterone system (RAAS), induces a cascade of deleterious responses that contribute to hypertension and cardiac remodeling. It stimulates the release of norepinephrine and endothelin-1, thereby enhancing sympathetic tone and vasoconstriction, while concurrently promoting aldosterone secretion, which leads to sodium retention, volume overload, and myocardial fibrosis. The figure also emphasizes angiotensin II’s capacity to degrade bradykinin, reduce nitric oxide and prostaglandin production, and promote oxidative stress and endothelial dysfunction [118]. These processes collectively increase arterial stiffness, promote vascular smooth muscle cell proliferation, enhance leukocyte adhesion, and facilitate structural remodeling of the myocardium.

In contrast, the ACE inhibition panel (orange box) delineates the therapeutic blockade of these pathological mechanisms. ACE inhibitors prevent the conversion of angiotensin I to angiotensin II, attenuate vasoconstriction, and restore endothelial homeostasis. Moreover, by preserving bradykinin levels, ACE inhibitors enhance the production of nitric oxide and prostacyclin, promoting vasodilation and antithrombotic effects. The downstream outcomes include reduced vascular resistance, decreased afterload, and inhibition of cardiac hypertrophy and fibrosis, the mechanistic foundations for their established role in managing heart failure and hypertension [119].

### 5.2. Challenges in the Use of Toxins as Cardiovascular Drugs

For over half a century, marine toxins and venoms have been recognized as a rich source of biologically active compounds with highly specific mechanisms of action. Since the 1960s, compounds such as tetrodotoxin and saxitoxin have been extensively studied for their ability to modulate ion channels with exceptional selectivity, leading to their use as pharmacological tools and templates for drug development. Cardiovascular research has shown that several peptides and small molecules derived from marine and terrestrial venoms exhibit promising cardioprotective properties, such as anti-arrhythmic effects, protection against ischemia–reperfusion injury, and ability to modulate blood pressure.

The use of toxins as cardioprotective agents presents both promising opportunities and significant obstacles. These compounds, often derived from marine organisms such as jellyfish, cone snails, or sponges, exhibit potent biological activities, including modulation of ion channels and strong anti-inflammatory effects. Despite their therapeutic potential, their inherent toxicity poses serious safety concerns if not properly modified or controlled. One major pharmacological challenge lies in their limited target specificity across tissue types. Although many marine toxins exhibit high molecular affinity due to their complex structures, they often lack functional selectivity when applied systemically, as they modulate ion channels or receptors that are also present in healthy, non-target tissues. This raises the risk of off-target effects such as unintended cardiac suppression or neurological symptoms, especially in cardiovascular applications where precise targeting is crucial [120].

Other obstacles in utilizing these toxic agents include narrow therapeutic windows, chemical instability in physiological environments, and difficulties in achieving safe drug formulation and targeted delivery. Additionally, the lack of clinical data and the risk of systemic side effects hinder their further development. Table 4 below lists several toxic marine compounds that have been investigated for cardioprotective effects, along with the associated obstacles and specific explanations. To date, the therapeutic exploration of marine-derived toxic compounds in cardiovascular medicine remains in its early stages.

However, the combination of unique structural complexity, high target affinity, and potent modulatory effects on key cardiovascular pathways, such as ion channels, neurohormonal regulation, and oxidative stress, positions these molecules as highly promising drug candidates. Several compounds, including ω-conotoxins, TTX, and conantokin-G, have already demonstrated selective pharmacological activity relevant to arrhythmia control, blood pressure regulation, and ischemia–reperfusion injury in preclinical models.

Moreover, the rich biodiversity of marine ecosystems offers an untapped reservoir of chemically diverse compounds, many of which have yet to be fully characterized or screened for cardiovascular activity. Advances in peptide engineering, targeted drug delivery, and structure-guided design are increasingly enabling the refinement of toxic peptides into safer, more selective therapeutic agents. Collectively, these factors highlight the significant potential of marine toxins as a novel class of cardioprotective therapeutics, particularly for conditions where conventional drugs are insufficient or nonselective.

## 6. Concluding Remark and Future Direction

Marine-derived toxins and venoms demonstrate substantial potential as cardioprotective agents due to their capacity to modulate ion channel function, mitigate oxidative stress, and prevent myocardial injury. Bioactive compounds such as tetrodotoxin, ω-conotoxin, anthopleurin-A and -B, palytoxin, brevetoxin, aplysiatoxin, and asterosaponin exhibit diverse pharmacological activities relevant to cardiovascular protection. Nevertheless, their translation into clinical practice remains constrained by inherent challenges, particularly their high toxicity and narrow therapeutic indices. Consequently, a detailed risk-benefit analysis is imperative to clearly distinguish therapeutic efficacy from potential toxicity. Future efforts must focus on defining precise safety margins, refining structural characteristics to enhance selectivity, and developing advanced delivery systems to minimize adverse effects.

Future research should incorporate sustainable biotechnological approaches to support environmentally responsible utilization of marine bioresources. The adoption of microalgae and cyanobacteria as alternative biosynthetic platforms offers a renewable and scalable approach to producing marine-derived bioactive compounds through blue biotechnology and metabolic engineering frameworks. Integrating sustainability principles with pharmacological innovation will not only facilitate the ethical and efficient production of these compounds but also strengthen the long-term potential of marine-derived cardioprotective agents in modern therapeutic development.

## Figures and Tables

**Table 1:** Representative examples of marine toxins along with their biochemical classes and lethal dose.

Compound	Biochemical Class	Chemical Polarity	Lethal Dose (LD_50_)	References
Domoic Acid (DA)	Amino acid derivative	Hydrophilic	2.5 mg/kg (i.p., rat)	[15,16]
ATX II	Protein	Hydrophilic	25–50 ng/animal (i.c.v., mouse)	[17,18]
ω-Conotoxin	Peptide	Hydrophilic	232 µg/kg (oral, mouse)	[19,20]
Saxitoxin (STX)	Alkaloid	Hydrophilic	1 µg/kg (i.p., mouse)	[21,22]
Tetrodotoxin (TTX)	Alkaloid	Hydrophilic	0.77 µg/kg (i.p., mouse)	[23,22]
Cyclic Imines (CI)	Alkaloid	Lipophilic	Not available	[24,25]
Okadaic Acid (OA)	Polyether	Lipophilic	192 µg/kg (i.p., mouse)	[26,27]
Ciguatoxin (CTX)	Polyether	Lipophilic	P-CTX1B 0.32 µg/kg (i.p., mouse)	[28,29]
			CTX3C 1.6 µg/kg (i.p., mouse)	
Yessotoxin (YTX)	Polyether	Lipophilic	269–328 µg/kg (i.p., mouse)	[30,31]
Azaspiracid (AZA)	Polyether	Lipophilic	74 µg/kg (i.p., mouse)	[32,33]
Brevetoxin	Polyether	Lipophilic	875 µg/kg (i.p., mouse)	[34,35]
Pectenotoxin	Polyketide	Lipophilic	219 µg/kg (i.p., mouse)	[36,37]
Palytoxin (PLTX)	Polyketide	Amphipathic	0.31–1.5 µg/kg (i.p., mouse)	[38,39]
Acanthaglycoside-G	Terpenoid	Amphipathic	Not available; toxicity only evaluated in vitro	[40]
Frondoside A	Saponin	Amphipathic	9.9 mg/kg (i.p., mouse)	[41,42]

**Table 2:** Types of toxic compounds with cardioprotective potential.

Compound	Source	Type	Distribution Region	Reference(s)
ω-conotoxin	*Conus magus*	Venom	Indo-Pacific (Philippines, Indonesia, PNG)	[52]
Proteins	*Conus striatus*	Venom	Indo-Pacific	[53]
Tetrodotoxin (TTX)	*Hapalochlaena* spp.	Toxin	Australia, Western Pacific, Japan	[54]
Alkaloid	*Takifugu rubripes*	Toxin	Northwest Pacific (China, Korea, Japan)	[55]
Alkaloid	*Takifugu porphyreus*	Hydrophilic		
Alkaloid	*Astropecten latespinosus*	Toxin	Western Pacific (Japan coastal regions)	[56]
Polyether	*Cephalothrix simula*	Toxin	Northwest Pacific	[57]
Polyether	*Thamnaconus modestus*	Toxin		[58]
Anthopleurin-A & B	*Anthopleura* spp.	Venom	Pacific and Atlantic coasts	[59]
Palytoxin (PLTXS)	*Palythoa* spp.	Toxin	Tropical Indo-Pacific, Hawaii, Red Sea	[60,61]
Brevetoxin	*Karenia brevis*	Toxin	Gulf of Mexico, Florida coast	[62,63,64]
Aplysiatoxin	*Cyanobacterium lyngbya*	Toxin	Indo-Pacific, Australia, Okinawa	[65,66]
Polyketide	*Nostoc muscorum*	Amphipathic	Indo-Pacific	[67]
Asterosaponin	*Acanthaster planci*	Toxin		[68,69]

**Table 3:** Status of toxic compounds being studied as cardiovascular drugs.

Compound	Source	Indication	Mechanism	Status	Trade Name	Approving Body	Reference(s)
ω-conotoxins	*Conus magus* (marine)	Ischemic heart injury, neuropathic pain	N-type CaV2.2 channel blocker	Under study	Prialt^®^	FDA	[93]
Conantokin-G	*Conus geographus* (marine)	Ischemic stroke, epilepsy	NMDA receptor antagonist	Preclinical	-	-	[93]
Tetrodotoxin (TTX)	*Takifugu* spp. (marine)	Ventricular arrhythmias, cancer pain	NaV channel blocker	Preclinical	-	CFDA/IND (USA)	[93,99]
Acanthaglycoside G	*Acanthaster planci* (marine)	Heart failure (potential)	Na^+^/K^+^-ATPase modulator	Under study	-	-	[81]
Anthopleurin-A & B	*Anthopleura* spp. (marine)	Heart failure	NaV channel sensitizer	Preclinical	-	-	[100,101,102]
Verrucotoxin (VTX)	*Synanceia verrucosa* (marine)	Cardioprotective in ischemia models	Modulates CaV and K_ATP_ channels	Under study	-	-	[103]
Goniopora toxin (GPT)	*Goniopora coral* (marine)	Positive inotropy	Prolongs cardiac action potential (NaV inactivation)	Preclinical	-	-	[104,105]

**Table 4:** Obstacles in the use of marine toxic compounds as cardioprotective agents.

Compound	Type	Limitation	Status	Explanation	Reference
Tetrodotoxin (TTX)	Toxin	High toxicity	Preclinical	TTX is highly toxic even in small doses, which makes it difficult to use therapeutically without modification or safe formulation.	[121]
ω-conotoxin	Venom	Target selectivity	Preclinical	It has specific effects on N-type calcium channels but can affect the nervous system and cause unwanted side effects.	[122]
Anthopleurin-A & B	Venom	Stability and formulation	Preclinical	This peptide can undergo rapid degradation in the body so it needs special formulation to extend its half-life.	[102]
Palytoxin (PLTX)	Toxin	Narrow therapeutic range	Preclinical	PLTX is one of the most toxic compounds in the world so its therapeutic use requires further research into safe doses.	[123]
Brevetoxin	Toxin	Risk of neurotoxicity	Under study	Despite its beneficial sodium channel modulating effects, brevetoxin may also cause neurological disorders in humans.	[124]
Aplysiatoxin	Toxin	Proinflammatory activity	Preclinical	Since inflammation accelerates atherosclerosis and exacerbates myocardial ischemia–reperfusion injury, structural simplification (e.g., Aplog-1) is required to eliminate these cardiotoxic side effects.	[125]
Asterosaponin	Toxin	Mechanism of action not fully understood	Under study	More research is needed on its pharmacological effects and potential toxicity before it can be developed into a drug.	[126]

## Data Availability

No new data were created or analyzed in this study.

## Abbreviations

The following abbreviations are used in this manuscript:

CVD	Cardiovascular Disease
WHO	World Health Organization
ACE	Angiotensin-Converting Enzyme
CaV	Voltage-Gated Calcium Channel
NaV	Voltage-Gated Sodium Channel
ROS	Reactive Oxygen Species
PKC	Protein Kinase C
NMDA	N-Methyl-D-Aspartate
NMDAR	N-Methyl-D-Aspartate Receptor
NO	Nitric Oxide
ATPase	Adenosine Triphosphatase
FDA	Food and Drug Administration
CFDA	China Food and Drug Administration
IND	Investigational New Drug
RAAS	Renin-Angiotensin-Aldosterone System
SAR	Structure-Activity Relationship
BPP	Bradykinin-Potentiating Peptide
AMI	Acute Myocardial Infarction
